# Exercise modality‐dependent mitochondrial respiratory capacity in satellite cells and conditioned serum‐induced responses in cultured myotubes

**DOI:** 10.1113/EP092922

**Published:** 2025-06-13

**Authors:** Takanaga Shirai, Hayato Shinkai, Riku Tanimura, Kazuki Uemichi, Shunsuke Sugiyama, Kohei Takeda, Yu Kitaoka, Tohru Takemasa

**Affiliations:** ^1^ Department of Human Sciences Kanagawa University Kanagawa Japan; ^2^ Japan Society for Promotion Science Chiyoda‐ku Tokyo Japan; ^3^ Graduate School of Comprehensive Human Sciences University of Tsukuba Ibaraki Japan; ^4^ Research Organization of Science and Technology Ritsumeikan University Shiga Japan; ^5^ School of Political Science and Economic Meiji University Tokyo Japan; ^6^ Institute of Health and Sport Sciences University of Tsukuba Ibaraki Japan

**Keywords:** exercise conditioned serum, exercise modality, mitochondrial respiration, satellite cell, skeletal muscle

## Abstract

Exercise‐induced mitochondrial adaptations contribute to muscle function and metabolic health. We aimed to investigate the association of moderate‐intensity swimming (MOD) and high‐intensity interval training (HIIT) with mitochondrial function in skeletal muscle cells treated with exercise‐conditioned serum. Male ICR mice (7–8 weeks old) were assigned to the Sedentary, MOD or HIIT group. The MOD group underwent five sessions of 60 min. The HIIT group performed weighted high‐intensity swimming intervals. This study assessed mitochondrial enzyme activity in the plantaris muscle, mitochondrial respiratory capacity in isolated satellite cells, and mitochondrial function in C2C12 myotubes treated with exercise‐derived serum. Serum was obtained immediately and 24 h postexercise to assess acute effects and chronic adaptations, respectively. The MOD and HIIT groups demonstrated significantly increased muscle citrate synthase and cytochrome *c* oxidase activities compared with the Sedentary group, but with no significant differences between the MOD and HIIT groups. Satellite cells exhibited higher basal respiration, ATP production and maximal respiratory capacity in the MOD group than in the Sedentary and HIIT groups. Acute serum notably improved maximal mitochondrial respiration in cultured C2C12 myotubes in the HIIT group, whereas serum from chronic training improved those parameters but demonstrated no modality‐specific effects. MOD enhances mitochondrial respiratory function in satellite cells, probably owing to sustained aerobic metabolic signalling, whereas HIIT produces a potent but transient systemic response that acutely boosts mitochondrial function in muscle cells. The differential effects of exercise modalities emphasize the importance of timing and exercise modality in driving specific mitochondrial adaptations, thereby providing valuable insights for tailored exercise prescriptions for optimizing metabolic health.

## Abstract (highlights)

- **What is the central question of this study?**
  How do different exercise modalities differentially regulate mitochondrial function in skeletal muscle, satellite cells and systemically, via exercise‐conditioned serum? Additionally, how does the timing of serum collection (acute vs. chronic) influence mitochondrial adaptations in cultured muscle cells?
- **What is the main finding and its importance?**
  Exercise modality differentially influences mitochondrial adaptations in skeletal muscle, satellite cells and systemic endocrine signalling via serum factors. Chronic and acute exercise exert distinct effects on mitochondrial respiration, suggesting that both training frequency and intensity modulate metabolic adaptations. These findings highlight the complex interplay between direct muscular adaptations and systemic exerkine‐mediated signalling, providing valuable insights for optimizing exercise interventions.

## 1 INTRODUCTION

Exercise is a fundamental driver of health and athletic performance, but the biological mechanisms that underlie the effects of different types of exercise on the body remain an active area of research. The mitochondria, the energy centres of our cells, are important to these adaptations because they not only produce ATP but also regulate cellular metabolism, redox balance and muscle regeneration (Alway et al., 2023; Holloszy, 1967). Understanding how different exercise regimens alter mitochondrial function is crucial for optimizing athletic performance, improving metabolic health and developing effective exercise interventions (Jacobs & Lundby, 2013; Schytz et al., 2024).

Moderate‐intensity endurance exercises, such as running and swimming, and high‐intensity interval training (HIIT) represent two distinct approaches to physical conditioning (Gibala & McGee, 2008). Endurance exercise involves continuous, submaximal activity that enhances oxidative metabolism and increases mitochondrial content and efficiency over time (Holloszy, 1967). In contrast, HIIT consists of short bursts of exercise at an intensity close to or exceeding maximal oxygen uptake alternated with brief recovery periods, challenging both aerobic and anaerobic systems (Gibala et al., 2009; Tabata et al., 1997, 1996). HIIT is beneficial for developing metabolic flexibility, and it places substantial stress on the body, causing significant fluctuations in energy demands and metabolic byproducts, such as lactate (Bartlett et al., 2012; Pengam et al., 2023; Tamura et al., 2024). A gap remains in our understanding of how these exercise modalities differentially affect mitochondria, not only within skeletal muscle but also in satellite cells (the resident stem cells of the muscle that are crucial for growth and repair), despite extensive research (Fukuda et al., 2019; Lepper et al., 2011). Additionally, the effect of exercise‐induced factors circulating in the bloodstream, known as exerkines, adds another layer of complexity (Sato et al., 2022; Thyfault & Bergouignan, 2020). These exerkines, consisting of proteins, metabolites and extracellular vesicles, affect remote tissues and influence cellular metabolism. However, the specific mechanisms by which these systemic variables regulate mitochondrial function remain poorly understood (Shimazu et al., 2013; Valadi et al., 2007).

To address this gap, we used a serum‐addition model, in which cultured C2C12 myotubes were treated with serum obtained from exercised animals. This model provides a controlled method for investigating the potential systemic effects of exercise on mitochondrial function in vitro (Shirai et al., 2023; Shirai et al., 2023). Recent studies have demonstrated that systemic humoral factors can directly modulate skeletal muscle function, as seen in idiopathic inflammatory myopathies, where patient serum induces muscle weakness independent of immune cell infiltration (Leijding et al., 2024). We can better understand how blood‐conditioning signals, such as myokines, metabolites and other bioactive molecules, drive mitochondrial adaptations in muscle cells by isolating the effects of circulating factors from the direct mechanical and neural stimuli of exercise. Additionally, the serum‐addition approach enables comparison of the immediate and long‐term effects of exercise‐derived exerkines, providing insight into how the body communicates exercise‐induced benefits across different tissues (Nishikori et al., 2023; Sato et al., 2022).

In this study, we hypothesized that different exercise modalities [chronic moderate‐intensity swimming (MOD) and HIIT] would induce distinct mitochondrial adaptations, not only within skeletal muscle but also in satellite cells, and through systemic factors derived from serum after acute exercise or chronic training. Therefore, the aim of our study was to elucidate how exercise modality shapes mitochondrial function at multiple biological levels. To achieve this, we investigated mitochondrial function in three key contexts: (1) enzyme activity in skeletal muscle to evaluate local mitochondrial adaptations; (2) mitochondrial respiratory capacity in isolated satellite cells to examine the direct impact of exercise modality on muscle stem cells; and (3) the effects of exercise‐conditioned serum on mitochondrial function in cultured C2C12 myotubes to explore systemic influences.

## 2 MATERIALS AND METHODS

### 2.1 Ethical approval

All experimental procedures carried out in this study were based on the guidelines of National Institutes of Health (NIH) for Care and Use of Laboratory Animals (National Research Council Committee for the Update of the *Guide for the Use of Laboratory Animals*, 2011) and were authorized by the Institutional Animal Experiment Committee of the University of Tsukuba (animal ethical permission number: 23‐380).

### 2.2 Animals

Male Institute of Cancer Research (ICR) mice (7–8 weeks old, The Jackson Laboratory Japan Inc., Japan) were housed in controlled conditions [temperature, 22°C ± 2°C; humidity, 55% ± 5%; light–dark cycle: 12 h–12 h (lights on 07.00–19:00 h)], with ad libitum access to food and water. Only male mice were used to avoid variability owing to the oestrous cycle in females, which can influence metabolic and mitochondrial responses in skeletal muscle and blood (Aguiar et al., 2018; Reho et al., 2024; Rosa‐Caldwell et al., 2021). Mice were randomly assigned to three groups: Sedentary, MOD or HIIT. To assess long‐term adaptations, chronic training interventions were implemented in the MOD and HIIT groups. Chronic training was defined as a structured and repeated exercise protocol conducted five times per week for a duration of 4 weeks, aiming to induce sustained physiological and mitochondrial adaptations. The chronic training groups (*n* = 10 per group) underwent these repeated exercise sessions over the designated period. Blood and muscle samples were collected under general anaesthesia 24 h after the final exercise session to minimize acute metabolic alterations and to capture long‐term mitochondrial adaptations. As a result, chronic animals were sampled at 12 weeks of age. For acute exercise, high‐intensity intermittent exercise (HIIE) was defined as a single bout of high‐intensity exercise, whereas HIIT was a chronic intervention consisting of repeated high‐intensity exercise sessions over time. In the acute exercise groups (*n* = 8 per group), blood samples were collected immediately postexercise to examine transient metabolic and physiological responses. These animals were sampled at 8 weeks of age.

### 2.3 Blood and muscle issue collection

All blood samples were collected under general anaesthesia using 2% isoflurane inhalation (KN‐1701; Natsume, Tokyo, Japan), ensuring adequate sedation before the procedure. Blood was sampled from the inferior vena cava using a syringe and transferred to microtubes. Samples were allowed to clot at room temperature for 2 h, then centrifuged at 4°C (3000 r.p.m., 15 min) using a Tomy Digital Biology Co., Ltd centrifuge (Tokyo, Japan) to obtain serum. Each mouse yielded ∼1 mL of blood, with serum volumes averaging ∼500 µL. In chronic training groups, sampling was performed 24 h after the final session to minimize acute responses and reflect long‐term mitochondrial adaptations, whereas in acute exercise groups, samples were collected immediately postexercise to capture transient metabolic and physiological changes. In both groups, animals were killed under deep general anaesthesia via cervical dislocation in accordance with institutional and NIH guidelines for humane treatment.

Immediately post‐mortem, muscle tissues [specifically, the plantaris and extensor digitorum longus (EDL)] were carefully excised from the hindlimbs. The hindlimbs were secured in a supine position to ensure precise dissection. The plantaris muscle was isolated by making an incision along the posterior compartment of the lower hindlimb, separating it from the surrounding soleus and gastrocnemius muscles. The EDL muscle was dissected from the anterior compartment by exposing the tibialis anterior (TA) and removing the EDL along its natural anatomical plane. After excision, both the plantaris and EDL muscle samples were weighed immediately. The plantaris samples were then frozen in liquid nitrogen and stored at −80°C for subsequent analysis. The EDL muscle was used for satellite cell isolation through enzymatic digestion and cell extraction procedures.

### 2.4 Swimming exercise protocol

The MOD group underwent swim training five times per week for 4 weeks, with each session lasting 60 min. The exercise was conducted in a rectangular tank (42 cm × 64 cm × 38 cm) filled with water maintained at 37°C ± 1°C. In each session, all mice in the MOD group were placed in the tank simultaneously and exercised together to ensure consistency in training conditions (Takeda & Takemasa, 2015).

### 2.5 HIIE protocol

The HIIE protocol followed previously described methods (Abe et al., 2015; Shirai, Uemichi et al., 2023) and was performed five times per week for 4 weeks, like the MOD protocol. Unlike the MOD protocol, the HIIE protocol used a separate cylindrical barrel as a swimming pool, with water maintained at 37°C. Mice in the HIIE group swam with weights equivalent to 10% of their body weight attached to the proximal end of their tails. Each HIIE session consisted of 12 swimming sets or continued until exhaustion. Each set included 20 s of high‐intensity swimming followed by a 10 s rest period, during which the mice were removed from the water. Unlike the MOD protocol, where all mice swam together, the HIIE protocol was conducted with two mice at a time to allow precise monitoring of performance and fatigue. After completing the HIIE session, all mice had their fur dried using a Kim towel.

### 2.6 Enzyme activity

The maximal activities of citrate synthase (CS) and cytochrome *c* oxidase (COX) were measured in homogenates of the entire plantaris muscle. Specifically, a muscle portion (10–20 mg) was homogenized in 100 v/w of 100 mmol/L potassium phosphate buffer. Aliquots for CS enzyme activity were mixed with a reaction mixture containing (mM): 100 Tris, 1.0 DTNB, 3 acetyl‐CoA and 10 oxaloacetate (pH 8.0) in a 96‐well microplate. The absorbance changes were measured at 412 nm. Aliquots for COX enzyme activity were mixed with a reaction mixture containing (mM): 10 phosphate and 0.5 cytochrome *c* (reduced with sodium hydrosulphite, pH 7.0) in a 96‐well microplate. Alterations in absorbance were measured at 550 nm/min. CS and COX activities were identified spectrophotometrically using the methods described by Takahashi et al. (2024).

### 2.7 Measurement of blood lactate levels

Blood lactate levels were measured using a portable blood lactate analyser (Lactate Pro 2, Arkray, Japan). Blood samples were collected from the tail vein of mice before and after an immediate acute exercise intervention.

### 2.8 Cell culture

Murine C2C12 skeletal muscle cells (passage numbers 10–12) were seeded into six‐well plates and cultured in Dulbecco's modified Eagle's medium (DMEM; 11966025, Gibco) supplemented with 10% (v/v) fetal bovine serum (F9665, Sigma–Aldrich), 1% (v/v) penicillin (10 000 units/mL) and streptomycin (10 000 µg/mL) (15070‐063, Gibco), glucose (5 mM; G7021, Sigma–Aldrich), sodium pyruvate (1 mM) and GlutaMAX (1 mM; 35050‐038, Gibco) in a 5% CO_2_ humidified incubator at 37°C.

The satellite cells were collected from the EDL myofibre as described previously (Ono et al., 2012), with some modifications. The muscle sample, collected at 24 h after or immediately after exercise, was incubated in 0.2% collagenase (Worthington) dissolved in DMEM at 37°C for 90 min, with gentle agitation every 15 min, transferred to a dish containing DMEM, and separated using a wide‐mouthed Pasteur pipette five times. After incubation at 37°C for 1 h, growth medium was added, and the solution was plated into each well of a Matrigel‐coated six‐well plate. The cells were cultured for 120 h to promote cell proliferation. After sufficient proliferation, the cells were washed with DMEM, followed by the addition of trypsin solution. The cells were then transferred to a Matrigel (Corning)‐coated 10 cm culture dish and cultured until they reached 80%–90% confluency. Satellite cells were seeded at a density of 0.3 × 10^6^ cells per well in a six‐well plate. Satellite cells were cultured in GM (DMEM supplemented with 30% fetal bovine serum, 1% chicken embryo extract, 10 ng mL^−1^ fibroblast growth factor‐basic, and 1% penicillin–streptomycin) in a 5% CO_2_ humidified incubator at 37°C. The medium was changed to a differentiation medium, consisting of DMEM supplemented with 2% horse serum (Biowest, Nuaillé, France) and 1% non‐essential amino acids (Invitrogen), when the cells reached 90% confluence. This time point was day 0. The differentiation medium was changed every 24 h. The myotube medium was changed to an amino acid‐ and serum‐free medium (D9800‐13, United States Biological, Salem, MA, USA) (pH 7.3) on day 6 of differentiation in the serum‐supplemented experiment. They underwent a 1 h serum and amino acid starvation period pretreatment. The myotubes were subsequently treated with a medium containing 5% mouse serum for 24 h in each group. The serum doses used in previous studies ranged from 5% to 20% (Allen et al., 2021; Carson et al., 2018; Corrick et al., 2015). Thus, a 5% dilution was considered optimal for the experiments, considering the limited serum sample availability and the results of the preliminary experiments.

### 2.9 Seahorse XF96 extracellular flux measurement

A Seahorse XF96 Extracellular Flux Analyzer (Seahorse Bioscience, North Billerica, MA, USA) was used to evaluate the mitochondrial respiratory capacity. Isolated satellite cells or C2C12 myoblast cells were seeded into Seahorse XF96 plates at a density of 1.0 × 10^4^ cells per well. C2C12 myoblast cells were differentiated into myotubes as previously described. The sensor cartridges were hydrated 1 day before the experiment with an XF calibrate solution (pH 7.4) and incubated at 37°C in a non‐CO_2_ incubator for 24 h. Each well was washed with DMEM supplemented with 5.6 mM glucose, 1 mM sodium pyruvate, 32 mM NaCl and 2 mM GlutaMAX (pH 7.4) and incubated at 37°C for 60 min in serum‐ and amino acid‐free medium.

Baseline measurements of the oxygen consumption rate (OCR) were taken before sequential injection of the following inhibitors: 1 µM of oligomycin, which is an ATP synthase inhibitor; 2 µM of FCCP, which is a mitochondrial respiration uncoupler; and 1 µM of antimycin A and rotenone, which are mitochondrial electron transport blockers. Oligomycin was added initially, to estimate the proportion of basal OCR coupled to ATP synthesis.

### 2.10 Statistical analysis

Data were analysed using Graph Pad v.10.0 software (La Jolla, CA, USA), and the results are presented as means ± SD and individual values. All data were analysed using one‐way (group) or two‐way (group × time point) ANOVA. Statistical significance (*post hoc* test) was calculated based on Tukey's method when a significant *p*‐value was obtained. Statistical significance was set at *p*‐values of <0.05. Additionally, *post hoc* power analyses were conducted using G*Power 3.1 software (Heinrich Heine University, Düsseldorf, Germany) for key primary outcomes (CS and COX enzyme activity and maximal respiration). Effect sizes were calculated based on the observed group differences using one‐way ANOVA, and the statistical power (1 − β) for each parameter was determined. All analyses yielded power values of >0.80, supporting the sufficiency of the sample sizes used in this study.

## 3 RESULTS

### 3.1 Animal characteristics in skeletal muscles

**FIGURE 1:**
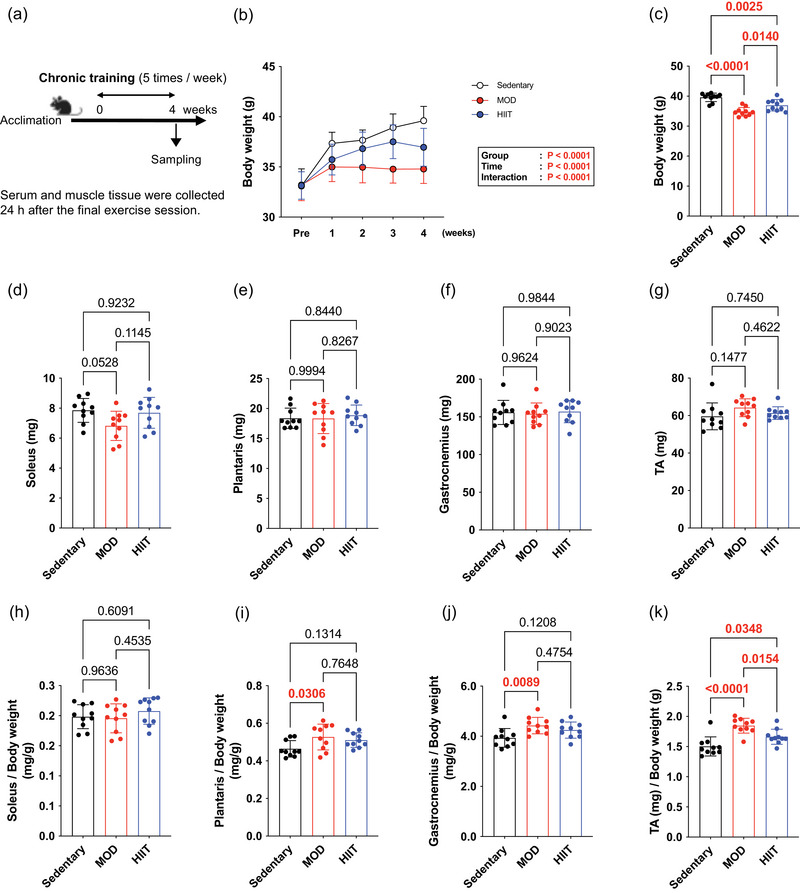
Experimental protocol and animal characteristics. (a) Experimental protocol. (b) Changes in body weight throughout the 4 week intervention. (c) Body weight. (d) Soleus wet weight. (e) Plantaris wet weight. (f) Gastrocnemius wet weight. (g) TA wet weight. (h) Soleus wet weight/body weight. (i) Plantaris wet weight/body weight. (j) Gastrocnemius wet weight/body weight. (k) TA wet weight/body weight. All data are expressed as means ± SD and individual values (*n* = 10). Significant differences were evaluated via ANOVA followed by Tukey's multiple comparison test. Significant differences were observed by connecting the corresponding groups with a line. Abbreviations: HIIE, high‐intensity intermittent exercise; MOD, moderate‐intensity swimming; TA, tibialis anterior.

Figure 1a shows a schematic representation of the experimental protocol. Both the MOD and HIIT groups demonstrated significant reductions in body weight compared with the Sedentary group after a 4 week intervention (main effect; group, *p* < 0.0001; time, *p* < 0.0001; interaction, *p* < 0.0001; Figure 1b). The MOD group exhibited significantly lower body weights than the Sedentary and HIIT groups (Sedentary, 39.6 ± 1.4 g; MOD, 34.8 ± 1.4 g; HIIT, 37.0 ± 1.9 g; Figure 1c). Wet weights of the soleus (Sedentary, 7.9 ± 0.8 mg; MOD, 6.8 ± 1.0 mg; HIIT, 7.7 ± 1.0 mg.), plantaris (Sedentary, 18.4 ± 1.7 mg; MOD, 18.3 ± 2.5 mg; HIIT, 18.9 ± 1.7 mg.), gastrocnemius (Sedentary, 155.8 ± 16.0 mg; MOD, 154.1 ± 14.3 mg; HIIT, 157 ± 14.7 mg) and TA (Sedentary, 59.6 ± 7.2 mg; MOD, 64.2 ± 4.8 mg; HIIT, 61.4 ± 3.4 mg) exhibited no significant difference between the groups (Figure 1d–g). The plantaris, gastrocnemius and TA muscle wet weights showed no significant differences among the groups (*p* > 0.05 for each comparison), indicating that the effects on muscle mass were specific to certain muscles. The plantaris, gastrocnemius and TA muscle wet weight to body weight ratios were significantly increased in the MOD group compared with the Sedentary group, but not in the HIIT group (Figure 1h–k). Both the MOD and HIIT regimens resulted in significant reductions in body weight compared with the Sedentary group, with the MOD group demonstrating the lowest final body weight. Swimming exercise exhibited a pronounced effect on muscle‐to‐body weight ratios, particularly affecting the plantaris, gastrocnemius and TA muscles. These results indicate distinct physiological adaptations induced by different exercise intensities, with the MOD group showing more substantial effects on muscle composition and relative muscle mass compared with the HIIT group.

### 3.2 Mitochondrial‐associated enzyme activity

**FIGURE 2:**
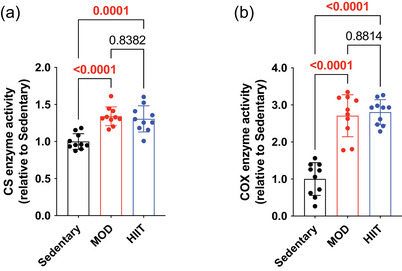
Mitochondrial‐associated enzyme activity in the plantaris muscle. (a) CS enzyme activity. (b) COX enzyme activity. All data are presented as means ± SD and individual values (*n* = 10). Significant differences were evaluated via one‐way ANOVA followed by Tukey's multiple comparison test. Significant differences were observed by connecting the corresponding groups with a line. Abbreviations: COX, cytochrome *c* oxidase; CS, citrate synthase; HIIE, high‐intensity intermittent exercise; MOD, moderate‐intensity swimming.

We assessed the effects of different exercise interventions (Sedentary, MOD and HIIT) on muscle CS and COX activities, which are key indicators of mitochondrial function. Both the MOD and HIIT groups demonstrated significant increases in CS (relative ratio: Sedentary, 1.0 ± 0.1; MOD, 1.3 ± 0.1; HIIT, 1.3 ± 0.2) and COX (relative ratio: Sedentary, 1.0 ± 0.5; MOD, 2.6 ± 0.6; HIIT, 2.8 ± 0.3) activity compared with the Sedentary group (Figure 2). These results indicate that both moderate‐ and high‐intensity exercise regimens improved mitochondrial enzyme function. However, no significant differences were observed in CS and COX activity between the MOD and HIIT groups, indicating that the exercise modality did not differentially affect mitochondria‐associated enzyme activity in skeletal muscle.

### 3.3 Effect of serum from mice undergoing chronic training on C2C12 myotubes

**FIGURE 3:**
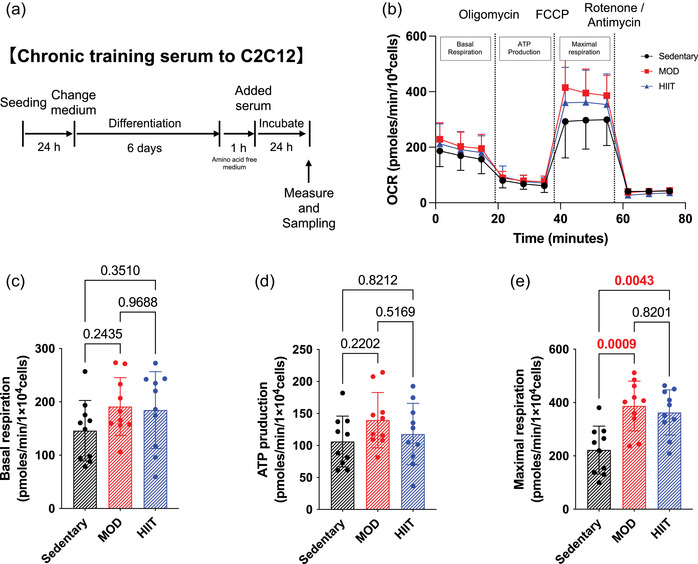
Difference of exercise modalities in chronic training serum on respiratory capacity in C2C12 muscle cells. (a) Experimental protocol in vitro. (b) OCR. (c) Basal respiration. (d) ATP production. (e) Maximal respiration. All data are presented as means ± SD and individual values (*n* = 10). Significant differences were evaluated via one‐way ANOVA followed by Tukey's multiple comparison test. Significant differences were observed by connecting the corresponding groups with a line. Abbreviations: HIIE, high‐intensity intermittent exercise; MOD, moderate‐intensity swimming; OCR, oxygen consumption rate.

**FIGURE 4:**
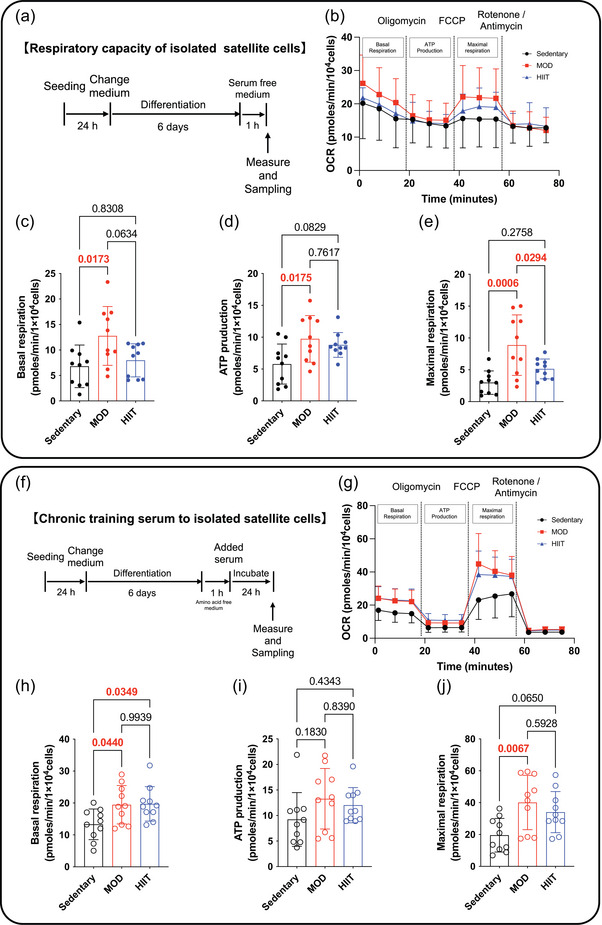
Difference of exercise modality on the respiratory capacity of isolated satellite cell itself and the effects of exercise‐conditioned serum. (a, f) Experimental protocol in vitro. (b, g) OCR. (c, h) Basal respiration. (d, i) ATP production. (e, j) Maximal respiration. All data are presented as means ± SD and individual values (*n* = 10). Significant differences were evaluated via one‐way ANOVA followed by Tukey's multiple comparison test. Significant differences were observed by connecting the corresponding groups with a line. Abbreviations: HIIE, high‐intensity intermittent exercise; MOD, moderate‐intensity swimming; OCR, oxygen consumption rate.

C2C12 myotubes were cultured in a medium containing 5% serum from exercised mice for 24 h in a serum‐supplementing model (Figure 3a). Figure 4b illustrates the overall OCR profile. These parameters of basal respiration and ATP production did not change in all groups (Figure 3c, d), but serum from the two exercise groups significantly increased maximal respiration compared with the Sedentary group (Figure 3e). These results indicate that serum from chronically trained mice increased the maximal respiration of cultured cells, but did not differ by its modalities.

### 3.4 Mitochondrial respiratory capacity of isolated satellite cells and the effects of exercise‐conditioned serum

Figure 4 illustrates the results of assessing mitochondrial respiratory capacity in satellite cells isolated from the skeletal muscle of mice in the Sedentary, MOD and HIIT groups. Mitochondrial function was measured using a cellular flux analyser to determine OCR, with a focus on basal respiration, ATP production‐linked respiration and maximal respiration. Figure 4a shows the in vitro experimental protocol. Figure 4b exhibits the overall OCR profile, which captures different phases of mitochondrial respiration, including basal respiration, ATP production and maximal respiration. Satellite cells in the MOD group significantly increased basal respiration and ATP production compared with those in the Sedentary group (*p* = 0.0173 and *p* = 0.0175, respectively) and maximal respiration compared with those in the MOD and HIIT groups, indicating improved mitochondrial activity at rest caused by MOD (Figure 4c–e). Our results revealed that satellite cells from the skeletal muscle of the MOD group exhibit significant improvements in basal respiration, ATP production‐related respiration and maximal respiration compared with the Sedentary group. In contrast, the HIIT group exhibited no significant increases in any of these mitochondrial respiration parameters. These results indicate that MOD is more effective than HIIT in enhancing mitochondrial respiratory function in satellite cells.

Next, we examined the effects of serum supplementation on isolated satellite cells. The serum supplementation protocol was performed in the same manner as the protocol used for C2C12 cells in Figure 3, and the OCR parameters are presented in Figure 4g. Basal respiration was significantly higher in the MOD and HIIT groups compared with the Sedentary group (Figure 4h). No significant differences were observed in ATP production among the groups (Figure 4i). Maximal respiration was significantly higher in the MOD group compared with both the Sedentary and HIIT groups (*p* = 0.0067, *p* = 0.0650; Figure 4j). These findings suggest that MOD enhances mitochondrial respiratory function in satellite cells more effectively than HIIT.

### 3.5 Serum from mice undergoing acute exercise to cultured skeletal muscle cells

**FIGURE 5:**
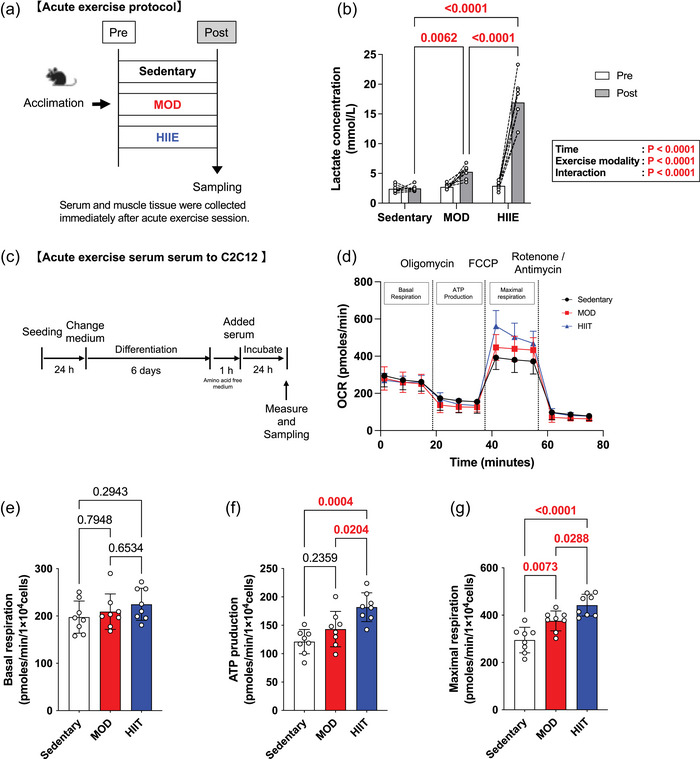
Difference of exercise modalities in effect of acute exercise serum on respiratory capacity in C2C12 muscle cells. (a) Acute exercise protocol. (b) Lactate concentration. (c) Experimental protocol in vitro. (d) OCR. (e) Basal respiration. (f) ATP production. (g) Maximal respiration. All data are presented as means ± SD and individual values (*n* = 8). Significant differences were evaluated via two‐way or one‐way ANOVA followed by Tukey's multiple comparison test. Significant differences were observed by connecting the corresponding groups with a line. Abbreviations: HIIE, high‐intensity intermittent exercise; MOD, moderate‐intensity swimming; OCR, oxygen consumption rate.

The study design of the acute exercise is shown in Figure 5a. The results demonstrate that blood lactate levels increased significantly following acute exercise interventions. Both the MOD and HIIE groups exhibited markedly elevated lactate concentrations compared with the Sedentary group (main effect of exercise, *p* < 0.0001). Furthermore, blood lactate levels were significantly elevated in the MOD group compared with the Sedentary group. Moreover, lactate levels in the HIIE group were significantly higher than those in both the Sedentary and MOD groups (main effect of exercise modality, *p* < 0.0001; postexercise lactate concentration levels of Sedentary, 2.5 ± 0.48 mmol; MOD, 5.3 ± 0.48 mmol; HIIE, 16.9 ± 3.89 mmol), indicating that high‐intensity exercise induces a greater accumulation of blood lactate (Figure 5b). Similar to the cell culture protocol in Figure 4, we measured the respiratory capacity of cultured cells using serum from mice immediately after acute exercise. Serum from acutely exercised mice did not increase basal respiration in cultured cells as it did in chronically trained mice (Figure 5e). ATP production was significantly higher in the HIIT group compared with both the Sedentary and MOD groups, indicating a distinct metabolic adaptation induced by acute HIIE (Figure 5f). Interestingly, serum from the HIIE group significantly increased maximal respiration compared with the Sedentary and MOD groups (*p* < 0.0001 and *p* = 0.0288, respectively; Figure 5g). These results indicate that the serum immediately after acute exercise in mice undergoing HIIE exhibits increased mitochondrial maximal respiration.

## 4 DISCUSSION

Our study provides significant insight into how different exercise modalities (MOD and HIIT) produce distinct mitochondrial adaptations in skeletal muscle, satellite cells and systemic regulation through exercise‐derived serum. We examined critically the mechanisms underlying these adaptations, the impact of the timing of serum sampling, and the broader implications for exercise physiology. Although we did not directly quantify specific exerkines, our serum‐based approach captures a complex interplay of circulating factors beyond the effects of individual proteins.

Importantly, our findings indicate that exercise intensity modulates the acute serum response, with HIIT‐derived serum significantly enhancing ATP production and maximal respiration in muscle cells. This suggests that transient spikes in metabolic stress during high‐intensity exercise drive immediate mitochondrial adaptations. In contrast, the long‐term effects of chronic training appear to converge across exercise modalities, because no major differences were observed between the effects of chronic serum from moderate‐intensity and HIIT groups. This aligns with previous research indicating that sustained endurance exercise promotes a stable baseline shift in mitochondrial function, probably mediated by cumulative adaptations in systemic factors, such as myokine secretion, angiogenesis and oxidative enzyme activity. The systemic effects of chronic training suggest that prolonged exposure to exercise‐induced exerkines leads to more generalized enhancements in mitochondrial function, independent of training intensity. This supports the idea that endurance‐based exercise, regardless of intensity, can induce long‐term metabolic benefits through endocrine and paracrine signalling pathways. Future research should focus on identifying the temporal dynamics of exerkine secretion and their specific contributions to mitochondrial remodelling over time.

### 4.1 Effect of exercise modality on respiratory capacity of satellite cells

One of our most noteworthy findings is the enhanced mitochondrial respiratory capacity observed in satellite cells from the MOD group compared with those from the Sedentary and HIIT groups. The chronic MOD protocol appears to develop a metabolic environment that is highly conducive to mitochondrial biogenesis and efficiency (Ju et al., 2016). Regarding the mechanistic pathways in endurance exercise, swim training involves prolonged and continuous muscle contractions in aerobic conditions and robustly activates the AMPK and peroxisome proliferator‐activated receptor gamma coactivator 1‐alpha (PGC‐1α) signalling axes (Atherton et al., 2005; Baar et al., 2002). These pathways play a crucial role in mitochondrial biogenesis, upregulating genes involved in mitochondrial DNA replication, electron transport chain assembly and oxidative metabolism. Furthermore, AMPK also enhances the activity of sirtuins (e.g., SIRT1), which deacetylate and activate PGC‐1α, thereby amplifying mitochondrial gene transcription and promoting autophagy to maintain mitochondrial quality (Silvestre et al., 2014; Zeng et al., 2020). The steady‐state aerobic exercise inherent in swimming reduces the risk of excessive production of reactive oxygen species, instead facilitating a controlled, low‐level oxidative signal that promotes mitochondrial resilience and adaptability.

In contrast, HIIT causes high metabolic stress in satellite cells. Repeated, short bursts of maximal effort caused substantial hypoxia, lactate build‐up and spikes in reactive oxygen species, collectively stabilizing hypoxia‐inducible factors (Abe et al., 2015; Pescador et al., 2010; Semenza, 2010). This hypoxic response drives satellite cells towards a glycolytic phenotype, thereby limiting the extent of mitochondrial biogenesis (Jung et al., 2024; Kung‐Chun Chiu et al., 2019; Parra et al., 2017). Additionally, the high levels of reactive oxygen species developed during HIIT might affect mitochondrial integrity by damaging mitochondrial DNA and proteins, thereby diminishing respiratory capacity (Flockhart et al., 2021; Larsen et al., 2016). The oscillating energy demands of HIIT might not provide the sustained signals required for optimal mitochondrial adaptations in satellite cells, despite the activation of AMPK and related pathways.

Skeletal muscle satellite cell activation during regeneration from injury and after chronic exercise training is related to mitochondrial activation. Previous reports have revealed increased mitochondrial DNA content and ATP moles per cell in isolated satellite cells following pharmacological injury and chronic endurance training (Chen et al., 2020; Rodgers et al., 2014). Additionally, satellite cell proliferation after chronic denervation is associated with mitochondrial activation, according to the Mito‐tracker green staining level (Wong et al., 2021). A similar response revealed in this study indicates that increased mitochondrial respiratory function caused by chronic swimming is an indicator of satellite cell activation and proliferation. In contrast, Abreu and Kowaltowski (2020) revealed that a decrease in the OCR of isolated satellite cells after chronic endurance exercise mediates self‐renewal. The detailed mechanism explaining the discrepancy with our data is unclear, because the HIIT group demonstrated lower maximal respiration than the MOD group, but differences in exercise modalities or intensity might be a factor in identifying satellite cell dynamics after chronic exercise.

### 4.2 Effect of serum from chronically trained mice on C2C12

No significant difference in mitochondrial respiratory capacity was observed between the two modalities when C2C12 myotubes were exposed to serum collected 24 h postexercise from the MOD and HIIT groups. These results indicate that the systemic signals elicited by exercise become more generalized and that the acute specificity observed immediately after exercise is absent. The timing of serum collection demonstrates that the effect of the 24 h recovery period on serum composition is crucial. The acute metabolic and hormonal responses diminished 24 h after exercise, resulting in a recovery‐oriented serum profile (Ball, 2015; Kraemer & Ratamess, 2005; Sasaki et al., 2014). Exercise induces a dynamic network of exerkines, metabolites and extracellular vesicles, which are likely to contribute to the observed mitochondrial adaptations. This systemic response might involve key signalling molecules, such as lactate, interleukin‐6, fibroblast growth factor 21, brain‐derived neurotrophic factor and vascular endothelial growth factor (Chow et al., 2022; Thyfault & Bergouignan, 2020), whose combined and interactive effects cannot be explained fully by analysing single proteins alone. Rather than isolating specific molecules, our approach allows us to assess the physiological relevance of the cumulative systemic response in real time, providing a more integrative understanding of how exercise‐driven circulating factors modulate mitochondrial function.

### 4.3 Potent association of acute HIIT serum with myotube mitochondrial respiration

Serum collected immediately after HIIT demonstrated a pronounced effect on improving mitochondrial respiratory capacity in C2C12 myotubes, in contrast to the chronic training results. This effect emphasizes the potent but transient nature of the metabolic and hormonal surge caused by high‐intensity exercise. The serum is rich in stress hormones, such as catecholamines (adrenaline and noradrenaline), lactate and inflammatory cytokines, immediately after HIIT (Dorneles et al., 2016; Gosselin et al., 2012; Zhang et al., 2021). Lactate, once considered a mere metabolic byproduct, has now been recognized as a signalling molecule that drives mitochondrial biogenesis by activating PGC‐1α (Hashimoto et al., 2007; Takahashi et al., 2019, 2022). It associates glycolytic energy production with oxidative metabolism, thereby preparing muscle cells for future energy demands. The profound effects of HIIT serum on mitochondrial respiration are attributable to these acute, high‐intensity signals. However, these benefits can be short lived, and they require adequate recovery to prevent chronic metabolic stress. In contrast, serum from the MOD group, which was collected at the same time point, exhibited a lower concentration of these acute stress mediators, reflecting the less dramatic metabolic shifts related to moderate‐intensity exercise. Hence, the immediate postexercise serum from HIIT had a more potent, although transient, effect on mitochondrial respiration.

### 4.4 Importance of timing in mitochondrial adaptations

The differences in mitochondrial adaptations between chronic and acute exercise responses emphasize the importance of sampling timing. With chronic training (24 h recovery), the body has shifted from acute metabolic stress to recovery and adaptation. The serum is enriched with long‐term adaptive signals that promote mitochondrial health and muscle repair. These signals are less modality specific and reflect the overall response of the body to repeated exercise bouts. In contrast, serum collected immediately after exercise captures the peak metabolic and hormonal activity (Contrepois et al., 2020). Increased lactate, catecholamine and inflammatory cytokine levels induced rapid mitochondrial adaptations. These acute responses are more profound for HIIT because of its intensity, explaining the stronger effects of HIIT serum on cultured myotubes.

### 4.5 Practical implications and future research directions

The results have practical implications for developing exercise regimens tailored to specific health and performance goals. Moderate‐intensity endurance exercise is ideal for individuals seeking long‐term mitochondrial health and muscle regeneration capacity. It supports stable mitochondrial adaptations and minimizes oxidative damage. Conversely, HIIT boosts metabolic flexibility and anaerobic performance; however, it should be managed carefully to prevent chronic stress and ensure adequate recovery. Future research should focus on characterizing the temporal dynamics of release of exerkines and understanding how the different components (e.g., extracellular vesicles, microRNAs) affect mitochondrial function in different tissues. Moreover, investigating sex differences and genetic factors will provide a more personalized approach to exercise prescription, particularly for populations with metabolic disorders or age‐related muscle decline.

### 4.6 Limitations

This study has certain limitations that should be acknowledged. First, although a priori power analysis was not conducted, we performed *post hoc* power analyses for each primary outcome to validate the adequacy of our sample sizes. The results demonstrated that the achieved power exceeded 0.80 for all main outcome measures, including maximal mitochondrial respiration in C2C12 and satellite cells and the CS and COX enzyme activity in the plantaris muscle. These findings indicate that the sample sizes used in this study (*n* = 8–10 per group) were sufficient to detect biologically meaningful effects. Nonetheless, future studies should incorporate prospective power calculations to enhance statistical robustness and reproducibility.

Second, our study did not address muscle fibre‐type differences or genetic variability, which affect mitochondrial responses. Additionally, future studies should investigate how chronic versus acute exercise responses vary according to sex and age to gain a better understanding of the broad applicability of our findings.

A key limitation of this study is that mitochondrial function in excised skeletal muscle was evaluated only through enzyme activity measurements, which provide indirect insights into mitochondrial adaptations. High‐resolution respirometry, such as the Oroboros O2k high‐resolution respirometry system (Oroboros Instruments, Innsbruck, Austria), could offer a more direct assessment of mitochondrial respiratory capacity in intact muscle fibres and should be considered in future studies. Another limitation is the absence of functional and performance‐based assessments, such as muscle strength or exercise capacity, which would help to contextualize mitochondrial adaptations in terms of physiological outcomes. Additionally, enzyme activity measurements and satellite cell isolation were conducted on different muscle groups, potentially introducing variability. Future studies should aim to standardize these analyses within the same muscle to ensure greater consistency and comparability in assessing mitochondrial adaptations to different exercise modalities. Although *post hoc* power analyses supported our sample sizes, we still acknowledge the importance of prospective power calculations in future research to ensure reproducibility and broader generalizability.

## 5 CONCLUSION

In summary, this study shows that different exercise modalities are associated with varying mitochondrial responses in satellite cells and cultured myotubes treated with exercise‐conditioned serum. Moderate‐intensity exercise tended to enhance mitochondrial respiratory function in satellite cells, whereas serum derived from high‐intensity exercise increased mitochondrial respiration in C2C12 myotubes. These findings contribute to a better understanding of how exercise modality influences both cellular and systemic factors related to mitochondrial function.

## AUTHOR CONTRIBUTIONS

Takanaga Shirai, Kohei Takeda, Yu Kitaoka and Tohru Takemasa conceived and designed the research. Takanaga Shirai, Hayato Shinkai, Riku Tanimura, Kazuki Uemichi, Shunsuke Sugiyama, Kohei Takeda and Yu Kitaoka performed the experiment. Takanaga Shirai analysed data, interpreted the results of experiments, prepared the figures and wrote the manuscript. Takanaga Shirai, Hayato Shinkai, Kazuki Uemichi and Tohru Takemasa edited and revised the manuscript. All authors read and approved the manuscript and agree to be accountable for all aspects of the work in ensuring that questions related to the accuracy or integrity of any part of the work are appropriately investigated and resolved. All persons designated as authors qualify for authorship, and all those who qualify for authorship are listed.

## CONFLICT OF INTEREST

The authors declare no conflict of interest.

## Data Availability

All the data supporting the findings of the present study are available within the paper.

## References

[eph13894-bib-0001] Abe, T. , Kitaoka, Y. , Kikuchi, D. M. , Takeda, K. , Numata, O. , & Takemasa, T. (2015). High‐intensity interval training‐induced metabolic adaptation coupled with an increase in Hif‐1α and glycolytic protein expression. Journal of Applied Physiology (1985), 119(11), 1297–1302. 10.1152/japplphysiol.00499.2015 26429867

[eph13894-bib-0002] Abreu, P. , & Kowaltowski, A. J. (2020). Satellite cell self‐renewal in endurance exercise is mediated by inhibition of mitochondrial oxygen consumption. Journal of Cachexia, Sarcopenia and Muscle, 11(6), 1661–1676. 32748470 10.1002/jcsm.12601 PMC7749620

[eph13894-bib-0003] Aguiar, A. S., Jr. , Speck, A. E. , Amaral, I. M. , Canas, P. M. , & Cunha, R. A. (2018). The exercise sex gap and the impact of the estrous cycle on exercise performance in mice. Scientific Reports, 8(1), 10742. 30013130 10.1038/s41598-018-29050-0 PMC6048134

[eph13894-bib-0004] Allen, S. L. , Marshall, R. N. , Edwards, S. J. , Lord, J. M. , Lavery, G. G. , & Breen, L. (2021). The effect of young and old ex vivo human serum on cellular protein synthesis and growth in an in vitro model of aging. American Journal of Physiology‐Cell Physiology, 321(1), C26–C37. 33909501 10.1152/ajpcell.00093.2021

[eph13894-bib-0005] Alway, S. E. , Paez, H. G. , Pitzer, C. R. , Ferrandi, P. J. , Khan, M. M. , Mohamed, J. S. , Carson, J. A. , & Deschenes, M. R. (2023). Mitochondria transplant therapy improves regeneration and restoration of injured skeletal muscle. Journal of Cachexia, Sarcopenia and Muscle, 14(1), 493–507. 36604839 10.1002/jcsm.13153 PMC9891964

[eph13894-bib-0006] Atherton, P. J. , Babraj, J. , Smith, K. , Singh, J. , Rennie, M. J. , & Wackerhage, H. (2005). Selective activation of AMPK‐PGC‐1alpha or PKB‐TSC2‐mTOR signaling can explain specific adaptive responses to endurance or resistance training‐like electrical muscle stimulation. Federation of American Societies for Experimental Biology Journal, 19(7), 1–23. 15716393 10.1096/fj.04-2179fje

[eph13894-bib-0007] Baar, K. , Wende, A. R. , Jones, T. E. , Marison, M. , Nolte, L. A. , Chen, M. , Kelly, D. P. , & Holloszy, J. O. (2002). Adaptations of skeletal muscle to exercise: Rapid increase in the transcriptional coactivator PGC‐1. The Federation of American Societies for Experimental Biology Journal, 16(14), 1879–1886. 12468452 10.1096/fj.02-0367com

[eph13894-bib-0008] Ball, D. (2015). Metabolic and endocrine response to exercise: Sympathoadrenal integration with skeletal muscle. Journal of Endocrinology, 224(2), R79–R95. 25431226 10.1530/JOE-14-0408

[eph13894-bib-0009] Bartlett, J. D. , Hwa Joo, C. , Jeong, T. S. , Louhelainen, J. , Cochran, A. J. , Gibala, M. J. , Gregson, W. , Close, G. L. , Drust, B. , & Morton, J. P. (2012). Matched work high‐intensity interval and continuous running induce similar increases in PGC‐1α mRNA, AMPK, p38, and p53 phosphorylation in human skeletal muscle. Journal of Applied Physiology (1985), 112(7), 1135–1143. 10.1152/japplphysiol.01040.2011 22267390

[eph13894-bib-0010] Carson, B. P. , Patel, B. , Amigo‐Benavent, M. , Pauk, M. , Kumar Gujulla, S. , Murphy, S. M. , Kiely, P. A. , & Jakeman, P. M. (2018). Regulation of muscle protein synthesis in an in vitro cell model using ex vivo human serum. Experimental Physiology, 103(6), 783–789. 29607575 10.1113/EP086860

[eph13894-bib-0011] Chen, Z. , Li, L. , Wu, W. , Liu, Z. , Huang, Y. , Yang, L. , Luo, Q. , Chen, J. , Hou, Y. , & Song, G. (2020). Exercise protects proliferative muscle satellite cells against exhaustion via the Igfbp7‐Akt‐mTOR axis. Theranostics, 10(14), 6448–6466. 32483463 10.7150/thno.43577 PMC7255041

[eph13894-bib-0012] Chow, L. S. , Gerszten, R. E. , Taylor, J. M. , Pedersen, B. K. , van Praag, H. , Trappe, S. , Febbraio, M. A. , Galis, Z. S. , Gao, Y. , Haus, J. M. , Lanza, I. R. , Lavie, C. J. , Lee, C.‐H. , Lucia, A. , Moro, C. , Pandey, A. , Robbins, J. M. , Stanford, K. I. , Thackray, A. E. , … Snyder, M. P. (2022). Exerkines in health, resilience and disease. Nature Reviews Endocrinology, 18(5), 273–289. 10.1038/s41574-022-00641-2 PMC9554896 35304603

[eph13894-bib-0013] Contrepois, K. , Wu, S. , Moneghetti, K. J. , Hornburg, D. , Ahadi, S. , Tsai, M.‐S. , Metwally, A. A. , Wei, E. , Lee‐McMullen, B. , Quijada, J. V. , Chen, S. , Christle, J. W. , Ellenberger, M. , Balliu, B. , Taylor, S. , Durrant, M. G. , Knowles, D. A. , Choudhry, H. , Ashland, M. , … Snyder, M. P. (2020). Molecular choreography of acute exercise. Cell, 181(5), 1112–1130.e16. 32470399 10.1016/j.cell.2020.04.043 PMC7299174

[eph13894-bib-0014] Corrick, K. L. , Stec, M. J. , Merritt, E. K. , Windham, S. T. , Thomas, S. J. , Cross, J. M. , & Bamman, M. M. (2015). Serum from human burn victims impairs myogenesis and protein synthesis in primary myoblasts. Frontiers in Physiology, 6, 184. 26136691 10.3389/fphys.2015.00184 PMC4468386

[eph13894-bib-0015] Dorneles, G. P. , Haddad, D. O. , Fagundes, V. O. , Vargas, B. K. , Kloecker, A. , Romão, P. R. , & Peres, A. (2016). High intensity interval exercise decreases IL‐8 and enhances the immunomodulatory cytokine interleukin‐10 in lean and overweight‐obese individuals. Cytokine, 77, 1–9. 26476404 10.1016/j.cyto.2015.10.003

[eph13894-bib-0016] Flockhart, M. , Nilsson, L. C. , Tais, S. , Ekblom, B. , Apró, W. , & Larsen, F. J. (2021). Excessive exercise training causes mitochondrial functional impairment and decreases glucose tolerance in healthy volunteers. Cell Metabolism, 33(5), 957–970.e6. 33740420 10.1016/j.cmet.2021.02.017

[eph13894-bib-0017] Fukuda, S. , Kaneshige, A. , Kaji, T. , Noguchi, Y. T. , Takemoto, Y. , Zhang, L. , Tsujikawa, K. , Kokubo, H. , Uezumi, A. , Maehara, K. , Harada, A. , Ohkawa, Y. , & Fukada, S. I. (2019). Sustained expression of HeyL is critical for the proliferation of muscle stem cells in overloaded muscle. eLife, 8, e48284. 31545169 10.7554/eLife.48284 PMC6768661

[eph13894-bib-0018] Gibala, M. J. , & McGee, S. L. (2008). Metabolic adaptations to short‐term high‐intensity interval training: A little pain for a lot of gain?. Exercise and Sport Sciences Reviews, 36(2), 58–63. 18362686 10.1097/JES.0b013e318168ec1f

[eph13894-bib-0019] Gibala, M. J. , McGee, S. L. , Garnham, A. P. , Howlett, K. F. , Snow, R. J. , & Hargreaves, M. (2009). Brief intense interval exercise activates AMPK and p38 MAPK signaling and increases the expression of PGC‐1alpha in human skeletal muscle. Journal of Applied Physiology (1985), 106(3), 929–934. 10.1152/japplphysiol.90880.2008 19112161

[eph13894-bib-0020] Gosselin, L. E. , Kozlowski, K. F. , DeVinney‐Boymel, L. , & Hambridge, C. (2012). Metabolic response of different high‐intensity aerobic interval exercise protocols. Journal of Strength and Conditioning Research, 26(10), 2866–2871. 22124355 10.1519/JSC.0b013e318241e13d

[eph13894-bib-0021] Hashimoto, T. , Hussien, R. , Oommen, S. , Gohil, K. , & Brooks, G. A. (2007). Lactate sensitive transcription factor network in L6 cells: Activation of MCT1 and mitochondrial biogenesis. The Federation of American Societies for Experimental Biology Journal, 21(10), 2602–2612. 17395833 10.1096/fj.07-8174com

[eph13894-bib-0022] Holloszy, J. O. (1967). Biochemical adaptations in muscle. Effects of exercise on mitochondrial oxygen uptake and respiratory enzyme activity in skeletal muscle. Journal of Biological Chemistry, 242(9), 2278–2282. 4290225

[eph13894-bib-0023] Jacobs, R. A. , & Lundby, C. (2013). Mitochondria express enhanced quality as well as quantity in association with aerobic fitness across recreationally active individuals up to elite athletes. Journal of Applied Physiology (1985), 114(3), 344–350. 10.1152/japplphysiol.01081.2012 23221957

[eph13894-bib-0024] Ju, J. S. , Jeon, S. I. , Park, J. Y. , Lee, J. Y. , Lee, S. C. , Cho, K. J. , & Jeong, J. M. (2016). Autophagy plays a role in skeletal muscle mitochondrial biogenesis in an endurance exercise‐trained condition. Journal of Physiological Sciences, 66(5), 417–430. 10.1007/s12576-016-0440-9 PMC10716990 26943341

[eph13894-bib-0025] Jung, U. , Kim, M. , Dowker‐Key, P. , Noë, S. , Bettaieb, A. , Shepherd, E. , & Voy, B. (2024). Hypoxia promotes proliferation and inhibits myogenesis in broiler satellite cells. Poultry Science, 103(1), 103203. 10.1016/j.psj.2023.103203 PMC10685027 37980759

[eph13894-bib-0026] Kraemer, W. J. , & Ratamess, N. A. (2005). Hormonal responses and adaptations to resistance exercise and training. Sports Medicine, 35(4), 339–361. 15831061 10.2165/00007256-200535040-00004

[eph13894-bib-0027] Kung‐Chun Chiu, D. , Pui‐Wah Tse, A. , Law, C.‐T. , Ming‐Jing Xu, I. , Lee, D. , Chen, M. , Kit‐Ho Lai, R. , Wai‐Hin Yuen, V. , Wing‐Sum Cheu, J. , Wai‐Hung Ho, D. , Wong, C.‐M. , Zhang, H. , Ng, I. O.‐L. , & Chak‐Lui Wong, C. (2019). Hypoxia regulates the mitochondrial activity of hepatocellular carcinoma cells through HIF/HEY1/PINK1 pathway. Cell Death & Disease, 10, 934. 31819034 10.1038/s41419-019-2155-3 PMC6901483

[eph13894-bib-0028] Larsen, F. J. , Schiffer, T. A. , Ørtenblad, N. , Zinner, C. , Morales‐Alamo, D. , Willis, S. J. , Calbet, J. A. , Holmberg, H.‐C. , & Boushel, R. (2016). High‐intensity sprint training inhibits mitochondrial respiration through aconitase inactivation. The Federation of American Societies for Experimental Biology Journal, 30(1), 417–427. 26452378 10.1096/fj.15-276857

[eph13894-bib-0029] Leijding, C. , Kaewin, S. , Andreasson, K. M. , Horuluoglu, B. , Galindo‐Feria, A. S. , Van Gompel, E. , Dastmalchi, M. , Gastaldello, S. , Alexanderson, H. , Lundberg, I. E. , & Andersson, D. C. (2024). Serum from patients with idiopathic inflammatory myopathy induces skeletal muscle weakness. Annals of the Rheumatic Diseases, 83(12), 1796–1797. 39197873 10.1136/ard-2024-225912

[eph13894-bib-0030] Lepper, C. , Partridge, T. A. , & Fan, C. M. (2011). An absolute requirement for Pax7‐positive satellite cells in acute injury‐induced skeletal muscle regeneration. Development, 138(17), 3639–3646. 21828092 10.1242/dev.067595 PMC3152922

[eph13894-bib-0031] Nishikori, S. , Yasuda, J. , Murata, K. , Takegaki, J. , Harada, Y. , Shirai, Y. , & Fujita, S. (2023). Resistance training rejuvenates aging skin by reducing circulating inflammatory factors and enhancing dermal extracellular matrices. Scientific Reports, 13(1), 10214. 37353523 10.1038/s41598-023-37207-9 PMC10290068

[eph13894-bib-0032] Ono, Y. , Masuda, S. , Nam, H. S. , Benezra, R. , Miyagoe‐Suzuki, Y. , & Takeda, S. (2012). Slow‐dividing satellite cells retain long‐term self‐renewal ability in adult muscle. Journal of Cell Science, 125(5), 1309–1317. 22349695 10.1242/jcs.096198

[eph13894-bib-0033] Parra, V. , Bravo‐Sagua, R. , Norambuena‐Soto, I. , Hernández‐Fuentes, C. P. , Gómez‐Contreras, A. G. , Verdejo, H. E. , Mellado, R. , Chiong, M. , Lavandero, S. , & Castro, P. F. (2017). Inhibition of mitochondrial fission prevents hypoxia‐induced metabolic shift and cellular proliferation of pulmonary arterial smooth muscle cells. Biochimica et Biophysica Acta (BBA)—Molecular Basis of Disease, 1863(11), 2891–2903. 28739174 10.1016/j.bbadis.2017.07.018

[eph13894-bib-0034] Pengam, M. , Goanvec, C. , Moisan, C. , Simon, B. , Albacète, G. , Féray, A. , Guernec, A. , & Amérand, A. (2023). Moderate intensity continuous versus high intensity interval training: Metabolic responses of slow and fast skeletal muscles in rat. PLoS ONE, 18(10), e0292225. 37792807 10.1371/journal.pone.0292225 PMC10550171

[eph13894-bib-0035] Pescador, N. , Villar, D. , Cifuentes, D. , Garcia‐Rocha, M. , Ortiz‐Barahona, A. , Vazquez, S. , Ordoñez, A. , Cuevas, Y. , Saez‐Morales, D. , Garcia‐Bermejo, M. L. , Landazuri, M. O. , Guinovart, J. , & del Peso, L. (2010). Hypoxia promotes glycogen accumulation through hypoxia inducible factor (HIF)‐mediated induction of glycogen synthase 1. PLoS ONE, 5(3), e9644. 20300197 10.1371/journal.pone.0009644 PMC2837373

[eph13894-bib-0036] Reho, J. J. , Muskus, P. C. , Bennett, D. M. , Grobe, C. C. , Burnett, C. M. L. , Nakagawa, P. , Segar, J. L. , Sigmund, C. D. , & Grobe, J. L. (2024). Modulatory effects of estrous cycle on ingestive behaviors and energy balance in young adult C57BL/6J mice maintained on a phytoestrogen‐free diet. American Journal of Physiology‐Regulatory, Integrative and Comparative Physiology, 326(3), R242–R253. 38284128 10.1152/ajpregu.00273.2023 PMC11213288

[eph13894-bib-0037] Rodgers, J. T. , King, K. Y. , Brett, J. O. , Cromie, M. J. , Charville, G. W. , Maguire, K. K. , Brunson, C. , Mastey, N. , Liu, L. , Tsai, C. R. , Goodell, M. A. , & Rando, T. A. (2014). mTORC1 controls the adaptive transition of quiescent stem cells from G0 to G(Alert). Nature, 510(7505), 393–396. 24870234 10.1038/nature13255 PMC4065227

[eph13894-bib-0038] Rosa‐Caldwell, M. E. , Mortreux, M. , Kaiser, U. B. , Sung, D. M. , Bouxsein, M. L. , Dunlap, K. R. , Greene, N. P. , & Rutkove, S. B. (2021). The oestrous cycle and skeletal muscle atrophy: Investigations in rodent models of muscle loss. Experimental Physiology, 106(12), 2472–2488. 34569104 10.1113/EP089962 PMC8639792

[eph13894-bib-0039] Sasaki, H. , Morishima, T. , Hasegawa, Y. , Mori, A. , Ijichi, T. , Kurihara, T. , & Goto, K. (2014). 4 weeks of high‐intensity interval training does not alter the exercise‐induced growth hormone response in sedentary men. Springerplus, 3(1), 336. 25806146 10.1186/2193-1801-3-336 PMC4363223

[eph13894-bib-0040] Sato, S. , Dyar, K. A. , Treebak, J. T. , Jepsen, S. L. , Ehrlich, A. M. , Ashcroft, S. P. , Trost, K. , Kunzke, T. , Prade, V. M. , Small, L. , Basse, A. L. , Schönke, M. , Chen, S. , Samad, M. , Baldi, P. , Barrès, R. , Walch, A. , Moritz, T. , Holst, J. J. , … Sassone‐Corsi, P. (2022). Atlas of exercise metabolism reveals time‐dependent signatures of metabolic homeostasis. Cell Metabolism, 34(2), 329–345.e8. 35030324 10.1016/j.cmet.2021.12.016 PMC13189211

[eph13894-bib-0041] Schytz, C. T. , Ørtenblad, N. , Lundby, A. M. , Jacobs, R. A. , Nielsen, J. , & Lundby, C. (2024). Skeletal muscle mitochondria demonstrate similar respiration per cristae surface area independent of training status and sex in healthy humans. The Journal of Physiology, 602(1), 129–151. 38051639 10.1113/JP285091

[eph13894-bib-0042] Semenza, G. L. (2010). HIF‐1: Upstream and downstream of cancer metabolism. Current Opinion in Genetics & Development, 20(1), 51–56. 19942427 10.1016/j.gde.2009.10.009 PMC2822127

[eph13894-bib-0043] Shimazu, T. , Hirschey, M. D. , Newman, J. , He, W. , Shirakawa, K. , Le Moan, N. , Grueter, C. A. , Lim, H. , Saunders, L. R. , Stevens, R. D. , Newgard, C. B. , Farese, R. V., Jr. , de Cabo, R. , Ulrich, S. , Akassoglou, K. , & Verdin, E. (2013). Suppression of oxidative stress by β‐hydroxybutyrate, an endogenous histone deacetylase inhibitor. Science, 339(6116), 211–214. 23223453 10.1126/science.1227166 PMC3735349

[eph13894-bib-0044] Shirai, T. , Iwata, T. , Uemichi, K. , Tanimura, R. , Iwai, R. , & Takemasa, T. (2023). The effect of serum from calorie‐restricted mouse on mTOR signaling in C2C12 myotubes. Journal of Endocrinology, 259(2), e230111. 37606077 10.1530/JOE-23-0111

[eph13894-bib-0045] Shirai, T. , Myoenzono, K. , Kawai, E. , Yamauchi, Y. , Suzuki, K. , Maeda, S. , Takagi, H. , & Takemasa, T. (2023). Effects of maslinic acid supplementation on exercise‐induced inflammation and oxidative stress in water polo athletes: A randomized, double‐blind, crossover, and placebo‐controlled trial. Journal of the International Society of Sports Nutrition, 20(1), 2239196. 37498159 10.1080/15502783.2023.2239196 PMC10375926

[eph13894-bib-0046] Shirai, T. , Uemichi, K. , & Takemasa, T. (2023). Effects of the order of endurance and high‐intensity interval exercise in combined training on mouse skeletal muscle metabolism. American Journal of Physiology‐Regulatory, Integrative and Comparative Physiology, 325(5), R593–R603. 37746708 10.1152/ajpregu.00077.2023

[eph13894-bib-0047] Silvestre, M. F. , Viollet, B. , Caton, P. W. , Leclerc, J. , Sakakibara, I. , Foretz, M. , Holness, M. C. , & Sugden, M. C. (2014). The AMPK‐SIRT signaling network regulates glucose tolerance under calorie restriction conditions. Life Sciences, 100(1), 55–60. 24530742 10.1016/j.lfs.2014.01.080

[eph13894-bib-0048] Tabata, I. , Irisawa, K. , Kouzaki, M. , Nishimura, K. , Ogita, F. , & Miyachi, M. (1997). Metabolic profile of high intensity intermittent exercises. Medicine and Science in Sports and Exercise, 29(3), 390–395. 9139179 10.1097/00005768-199703000-00015

[eph13894-bib-0049] Tabata, I. , Nishimura, K. , Kouzaki, M. , Hirai, Y. , Ogita, F. , Miyachi, M. , & Yamamoto, K. (1996). Effects of moderate‐intensity endurance and high‐intensity intermittent training on anaerobic capacity and VO2max. Medicine and Science in Sports and Exercise, 28(10), 1327–1330. 8897392 10.1097/00005768-199610000-00018

[eph13894-bib-0050] Takahashi, K. , Kitaoka, Y. , & Hatta, H. (2024). Better maintenance of enzymatic capacity and higher levels of substrate transporter proteins in skeletal muscle of aging female mice. Applied Physiology, Nutrition and Metabolism, 49(8), 1100–1114. 10.1139/apnm-2024-0016 38710106

[eph13894-bib-0051] Takahashi, K. , Kitaoka, Y. , Matsunaga, Y. , & Hatta, H. (2019). Effects of lactate administration on mitochondrial enzyme activity and monocarboxylate transporters in mouse skeletal muscle. Physiological Reports, 7(17), e14224. 31512405 10.14814/phy2.14224 PMC6739509

[eph13894-bib-0052] Takahashi, K. , Tamura, Y. , Kitaoka, Y. , Matsunaga, Y. , & Hatta, H. (2022). Effects of lactate administration on mitochondrial respiratory function in mouse skeletal muscle. Frontiers in Physiology, 13, 920034. 35845998 10.3389/fphys.2022.920034 PMC9280083

[eph13894-bib-0053] Takeda, K. , & Takemasa, T. (2015). Expression of ammonia transporters Rhbg and Rhcg in mouse skeletal muscle and the effect of 6‐week training on these proteins. Physiological Reports, 3(10), e12596. 26471760 10.14814/phy2.12596 PMC4632962

[eph13894-bib-0054] Tamura, Y. , Jee, E. , Kouzaki, K. , Kotani, T. , & Nakazato, K. (2024). Monocarboxylate transporter 4 deficiency enhances high‐intensity interval training‐induced metabolic adaptations in skeletal muscle. The Journal of Physiology, 602(7), 1313–1340. 38513062 10.1113/JP285719

[eph13894-bib-0055] Thyfault, J. P. , & Bergouignan, A. (2020). Exercise and metabolic health: Beyond skeletal muscle. Diabetologia, 63(8), 1464–1474. 32529412 10.1007/s00125-020-05177-6 PMC7377236

[eph13894-bib-0056] Valadi, H. , Ekström, K. , Bossios, A. , Sjöstrand, M. , Lee, J. J. , & Lötvall, J. O. (2007). Exosome‐mediated transfer of mRNAs and microRNAs is a novel mechanism of genetic exchange between cells. Nature Cell Biology, 9(6), 654–659. 17486113 10.1038/ncb1596

[eph13894-bib-0057] Wong, A. , Garcia, S. M. , Tamaki, S. , Striedinger, K. , Barruet, E. , Hansen, S. L. , Young, D. M. , & Pomerantz, J. H. (2021). Satellite cell activation and retention of muscle regenerative potential after long‐term denervation. Stem Cells, 39(3), 331–344. 33326654 10.1002/stem.3316

[eph13894-bib-0058] Zeng, Z. , Liang, J. , Wu, L. , Zhang, H. , Lv, J. , & Chen, N. (2020). Exercise‐induced autophagy suppresses sarcopenia through Akt/mTOR and Akt/FoxO3a signal pathways and AMPK‐mediated mitochondrial quality control. Frontiers in Physiology, 11, 583478. 33224037 10.3389/fphys.2020.583478 PMC7667253

[eph13894-bib-0059] Zhang, H. , Tong, T. K. , Kong, Z. , Shi, Q. , Liu, Y. , & Nie, J. (2021). Exercise training‐induced visceral fat loss in obese women: The role of training intensity and modality. Scandinavian Journal of Medicine & Science in Sports, 31(1), 30–43. 32789898 10.1111/sms.13803

